# High-Frequency Chest Wall Oscillation in Patients with COVID-19: A Pilot Feasibility Study

**DOI:** 10.5152/eurasianjmed.2022.21048

**Published:** 2022-06-01

**Authors:** Mine Çelik, Ahmet Murat Yayık, Buğra Kerget, Ferhan Kerget, Ömer Doymuş, Alperen Aksakal, Sevilay Özmen, Mehtap Hülya Aslan, Yakup Uzun

**Affiliations:** 1 Department of Anesthesiology and Reanimation, Atatürk University Faculty of Medicine, Erzurum, Turkey; 2 Clinical Research, Development and Design Application and Research Center, Atatürk University Faculty of Medicine, Erzurum, Turkey; 3 Department of Pulmonary Diseases, Atatürk University Faculty of Medicine, Erzurum, Turkey; 4 Department of Infection Diseases and Clinical Microbiology, Erzurum Regional Education and Research Hospital, University of Health Sciences, Erzurum, Turkey; 5 Department of Anesthesiology and Reanimation, Erzurum Regional Education and Research Hospital, University of Health Sciences, Erzurum, Turkey; 6 Department of Pulmonary Diseases, Erzurum Regional Education and Research Hospital, University of Health Sciences, Erzurum, Turkey; 7 Department of Pathology, Atatürk University Faculty of Medicine, Erzurum, Turkey; 8 Department of Medical Microbiology, Erzurum Regional Education and Research Hospital, Erzurum, University of Health Sciences, Turkey; 9 Department of Mechanical Engineering, Atatürk University Faculty of Engineering, Erzurum, Turkey

**Keywords:** High-frequency chest wall oscillation, COVID-19, pneumonia

## Abstract

**Objective:** Coronavirus 2019 disease presents in a spectrum that can range from mild viral infection to pneumonia. Common symptoms of coronavirus disease 2019 pneumonia include cough, sputum, and shortness of breath. High-frequency chest wall oscillation is a pulmonary rehabilitation method used for the recovery of pulmonary functions and removal of secretions in the lungs. The aim of the study was to evaluate the efficacy of high-frequency chest wall oscillation on patients with coronavirus disease 2019 pneumonia.

**Materials and Methods:** In this study, 100 patients, between 18 and 70 years old, with a positive polymerase chain reaction result for coronavirus disease 2019, were included. Standard medical treatment was applied to all patients. In group rehabilitation, high-frequency chest wall oscillation treatment was applied twice a day for 20 minutes for 5 days. No additional intervention was made to the control group. Pulmonary function tests and oxygenation were evaluated on the first and fifth days. Patients’ high-flow oxygen, non-invasive mechanical ventilation, and invasive mechanical ventilation needs were evaluated and recorded.

**Results:** Compared with the control group, the forced expiratory volume in 1 second, forced vital capacity, and peak expiratory flow rates were statistically higher in the rehabilitation group on the fifth day (*P* < .05). On evaluating the oxygenation of patients, the fifth day to first-day oxygen saturation difference was significantly higher in rehabilitation group than in control group (*P* < .05). Furthermore, the number of patients who needed non-invasive mechanical ventilation was lower in the rehabilitation group (*P* < .05).

**Conclusion:** This study demonstrated that pulmonary rehabilitation applied with the high-frequency chest wall oscillation device in patients with coronavirus disease 2019 in the early period contributed to the improvement of oxygenation by providing significant improvement as observed in the pulmonary function tests of the patients.

## Introduction

On March 11, 2020, the World Health Organization (WHO) declared that the coronavirus disease 2019 (COVID-19) that started in Asia was a pandemic It was reported that the etiological agent was severe acute respiratory syndrome coronavirus 2, and the in-hospital mortality rate associated with COVID-19 was 5 times higher than influenza.^1^

According to the clinical classification of the WHO, COVID-19 disease presents in a spectrum that can range from mild disease to pneumonia, severe pneumonia, acute respiratory distress syndrome, sepsis, and septic shock.^2^ Common symptoms of this infection include fever, cough, sputum, and shortness of breath. Increased secretion and sputum plugs in the lungs disrupt gas exchange, cause atelectasis, and predispose individuals to secondary infections.^3,4^ This situation affects the response of the patients to treatment, duration of intensive care and hospital stay, and mortality.

Compared to conventional chest percussion and postdural drainage methods, high-frequency chest wall oscillation (HFCWO) is a pulmonary rehabilitation method used for the recovery of pulmonary functions and removal of secretions in the lungs.^5-8^ This method, which was originally used only in the management of cystic fibrosis, is now widely used in treating pulmonary, neurological, and neuromuscular diseases.^9,10^

Similar to other airway cleaning devices in the world that can provide HFCWO, Hamle©MED MEDİYES (Turkey) (medical vest systems) consists of an air pulse generator and a vest or chest strap. Using the air pulse generator, the system is filled with air and evacuated so that the rib cage is gently pressed and released up to a maximum of 20 times per second. High-frequency vibrations allow the excessive and thick pulmonary secretion accumulated in the airways to be thinned and easily coughed out. Pulmonary rehabilitation systems using the HFCWO device are used as an effective alternative to traditional chest physiotherapy in chronic lung diseases such as cystic fibrosis, chronic obstructive pulmonary disease (COPD), and bronchiectasis.^6,9,11^ This system acts on all lobes of the lungs simultaneously, independent of the patient’s position. It helps reduce pulmonary complications and increases clinical stability. Our study is the first study in the literature in which pulmonary physiotherapy method using HFCWO was utilized in patients with COVID-19.

The primary aim of the study was to evaluate the respiratory function tests (forced expiratory volume in 1 second (FEV1), forced vital capacity (FVC), FEV1/FVC, and peak expiratory flow (PEF)) performed on the first and fifth day of hospitalization on patients with COVID-19 treated with HFCWO. The secondary aims were to evaluate improvements in oxygenation, need for non-invasive mechanical ventilation (NIMV), invasive mechanical ventilation (IMV), high-flow oxygen (HFO), and length of hospital stay.

## Materials and Methods

The application for permission to ***The Republic of Turkey Ministry of the Health,*** Directorate General of Health Services Scientific Research was approved for this study. The study was conducted on patients with COVID-19 hospitalized in **Erzurum** hospitals after ethical approval was obtained from ***Atatürk University Faculty of Medicine*** Clinical Research Ethics Committee (decision no: 396).

Once written informed consent had been obtained from patients, we included 100 hospitalized patients in the ward during first hospitalization, without organ failure, and those who do not need intensive care, between the ages of 18 and 70 with a positive reverse transcriptase-polymerase chain reaction result for COVID-19 and pulmonary involvement. Patients with coronary artery disease, congestive heart failure, cardiac arrhythmia, bullous lung disease, chest trauma in the last month, and chest wall deformities were not included in the study.

The patients were randomly divided into 2 equal groups using Microsoft Office 365 Excel (Microsoft, Redmond, Wash, USA, http://www.microsoft.com) program. The rehabilitation group (group R, n = 50) consisted of patients who received pulmonary rehabilitation with HFCWO, whereas the control group (group C, n = 50) consisted of patients who did not receive HFCWO.

Medical treatment of all patients included in the study was organized according to the Ministry of Health Adult Patient Guide.^12^ Accordingly, following a loading dose of 1600 mg favipiravir twice a day, 600 mg twice a day maintenance treatment with the same drug was administered for 5-10 days. According to the risk status, the patients received anticoagulant treatment (enoxaparin sodium) in line with their body mass index. Methylprednisolone treatment at a dose of 1 mg/kg/day was administered to patients with moderate-to-severe pneumonia, and levofloxacin or moxifloxacin was administered as empirical antibiotherapy. Ceftriaxone and clarithromycin were administered to patients in whom respiratory tract quinolones could not be administered. During the follow-up, antibiotherapy was revised in line with the blood, urine, and sputum culture results.

**Figure 1.**
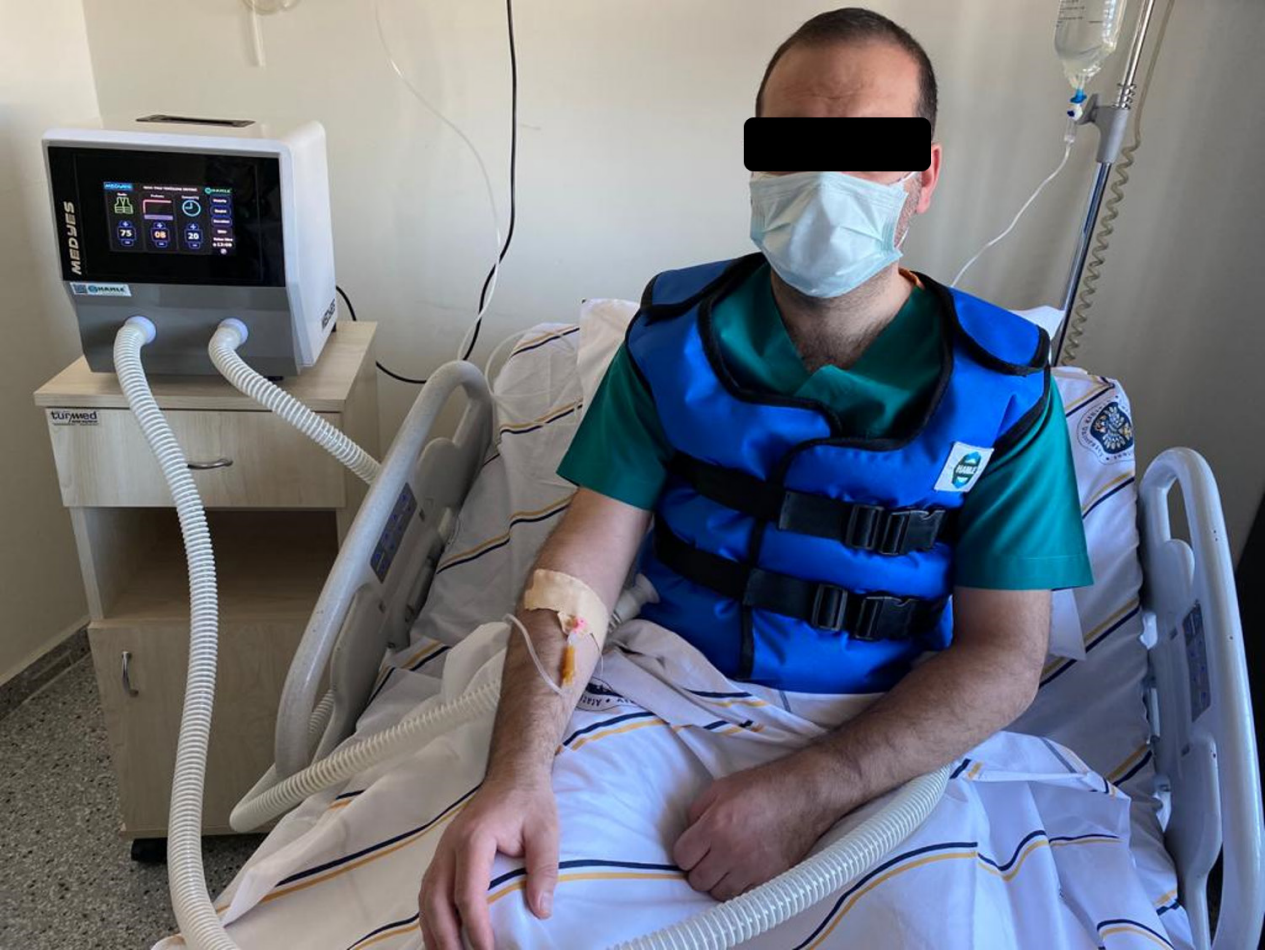
Application of high-frequency chest wall oscillation.

In Group R, HFCWO was applied in addition to COVID-19 medical treatment from the first day of admission for 5 days with Hamle©MED Mediyes (Turkey) HFCWO for approximately 20 minutes each in the morning and evening, in the presence of nurses trained in HFCWO. Pulmonary rehabilitation device settings were set to 75% pressure and 8 Hz frequency as standard for all patients (Figure 1). Rehabilitation therapy was performed with the patient alone in the room wearing a medical mask. After each rehabilitation session, the room was ventilated for 30 minutes with fresh air, and all surfaces were cleaned with surface disinfectant.

Laboratory tests used for COVID-19 diagnosis, treatment follow-up, and pulmonary function tests (PFTs) were performed on the first and fifth day of hospitalization in all patients.

### Pulmonary Function Tests

Pulmonary function tests were performed in a negative-pressure room by a technician wearing protective equipment to prevent transmission. The patients’ age, height, and weight were measured and recorded. Before testing, patients were instructed to abstain from smoking (for 24 hours), alcohol (for 4 hours), strenuous exercise (for 30 minutes), and heavy meals (for 2 hours). Tests were performed with the patients lightly dressed. Body temperature and pressure saturated correction was performed according to room air and barometric pressure. The technician explained the desired maneuvers to the patients, and they performed 3 acceptable spirometry analyses. As a result of this analysis, FEV1 (L), FVC (L), FEV1/FVC (%), and PEF (L/min) values were recorded. Tests that met the reproducibility and acceptability criteria were included in our analysis. All spirometry analyses were performed by the same technician using a Plusmed MIR Spirolab III device.

In addition, changes in oxygenation (room air and 2-4 L/min of oxygen by nasal cannula), need for HFO and NIMV, IMV, and exitus rates were recorded.

### Sputum Examination

Sputum samples taken after the third day of treatment were evaluated in terms of sputum cytology and sputum culture. Sputum collected in 70% alcohol in a suitable container was placed in a double bag (preferably with a hermetic seal or locked) for transport and stained with hematoxylin and eosin and Giemsa stains in a bio-safe environment, covered with a coverslip, and examined with a light microscope. In the cytomorphological examination of sputum, sputum in which bronchial epithelial cells and foamy histiocytes of alveoli were observed, were considered as the sufficient category, and sputum in which only squamous epithelial cells and elements of oropharyngeal flora were observed (squamous epithelial cells, *Candida* in oral mucosa, cocci, bacilli) were considered as insufficient.

In addition, sputum samples received at the microbiology laboratory were cultured on blood agar, eosin methylene blue agar, and chocolate agar media. Microbial growth was evaluated after being incubated for 24-48 hours at 37°C. For culture plates with growth, bacterial identification and antibiotic susceptibility tests were performed on the Phoenix automated system.

### Power analysis

A pilot study was conducted to determine the required sample size. The pilot study indicated that our primary parameter, forced vital capacity (FVC), was ~2.66 ± 0.82 L in the control group (n = 10) and 3.26 ± 0.82 L in the rehabilitation group (n = 10) on the fifth day. A total required sample size of 78 was calculated using GPower version 3.1.9.2 (Düsseldorf, Germany) with an alpha probability of 0.05, power of 0.90, and a large effect size of 0.74). Considering possible dropouts and to attain higher power, we decided to include at least 50 patients in each group.

### Statistical analysis

Statistical Package for the Social Sciences 22.0 (IBM SPSS Corp.; Armonk, NY, USA) software was used for statistical analysis. Pearson chi-square and Fisher’s exact test were used to compare categorical variables between groups. The normal distribution of numerical parameters was evaluated using Kolmogorov–Smirnov and histogram tests. The Student's *t*-test was used to compare normally distributed parameters and Mann–Whitney *U* test was used to compare parameters that were not normally distributed. Intragroup evaluations were made with the paired-samples *t*-test, and *P* values < .05 were considered statistically significant.

## Results

**Figure 2.**
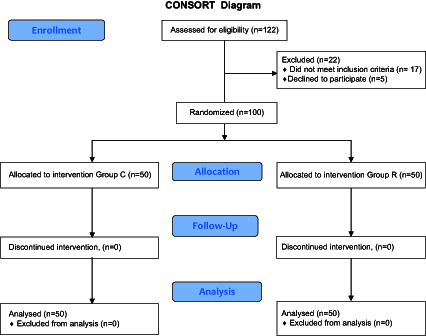
Consolidated standards of reporting trials.

Eligible patients for the study were analyzed for the primary outcomes. The flow diagram of the Consolidated Standards of Reporting Trials of the study is presented in Figure 2. There was no significant difference between the 2 groups in terms of demographic characteristics of the patients such as age, weight, height, sex, and history of chronic diseases, and there was no significant difference in terms of baseline characteristics of the patients (respiratory rate, heart rate, and blood pressure) (*P* > .05) (Table 1).

According to complete blood count results, there was no statistically significant difference in terms of white blood cells count, lymphocyte count, neutrophil count, hemoglobin, and platelet levels measured on the first and fifth day (*P* > .05) (Table 2). Further, there was no significant difference in terms of biochemical parameters in either group (*P* > .05) (Table 2).

In terms of oxygenation levels evaluation, there was no significant difference between groups on the first day and the fifth day (*P* > .05)**.** For intragroup evaluation, the fifth-day oxygenation was significantly higher in both group C and group R compared to the first day (*P* < .05) (Table 3). Fifth day to first-day oxygen saturation (SpO_2_) difference in the room air and 2-4 L/min oxygen was significantly higher in group R than group C (*P* < .05) (Table 4).

Pulmonary function tests were performed on the first and fifth day of hospitalization in all patients. None of the patients in both groups required intensive care and mechanical ventilation during this period. There was no significant difference between Group C and Group R on the first day in terms of the results of PFT evaluation, which is the primary aim of our study and is considered to be an indicator of improvement in the patient’s clinical condition, whereas there were significant differences in forced expiratory volume in 1 second (FEV1), forced vital capacity (FVC), and peak expiratory flow (PEF) rates between Group C and Group R on the fifth day (*P* < .05). Within the groups, there was no statistically significant difference in PFTs on the first and fifth days in Group C, whereas a statistically significant difference in the same was observed in Group R (*P* < .05) (Table 5).

None of the patients needed IMV, NIMV, and HFO initially during hospitalization. When the results in terms of need for HFO, NIMV, IMV, and exitus rates during hospital stay were compared, there was a significant difference between Group C and Group R in terms of NIMV need (*P* <0.05); however, no significant difference was found in terms of IMV need and exitus rates (*P* > .05). The need for HFO and IMV as well as mortality rates was considered in a clinical context in the control group, albeit not statistically significant. None of the patients in both groups required intensive care in the first 5 days of the treatment and no deaths occurred. However, 3 patients in the control group died on the 10th, 12th, and 14th days of the treatment. There was no significant difference between the 2 groups in terms of the length of hospital stay (*P* > .05) (Table 6).

### Cytopathological Examination

**Figure 3. a-d.**
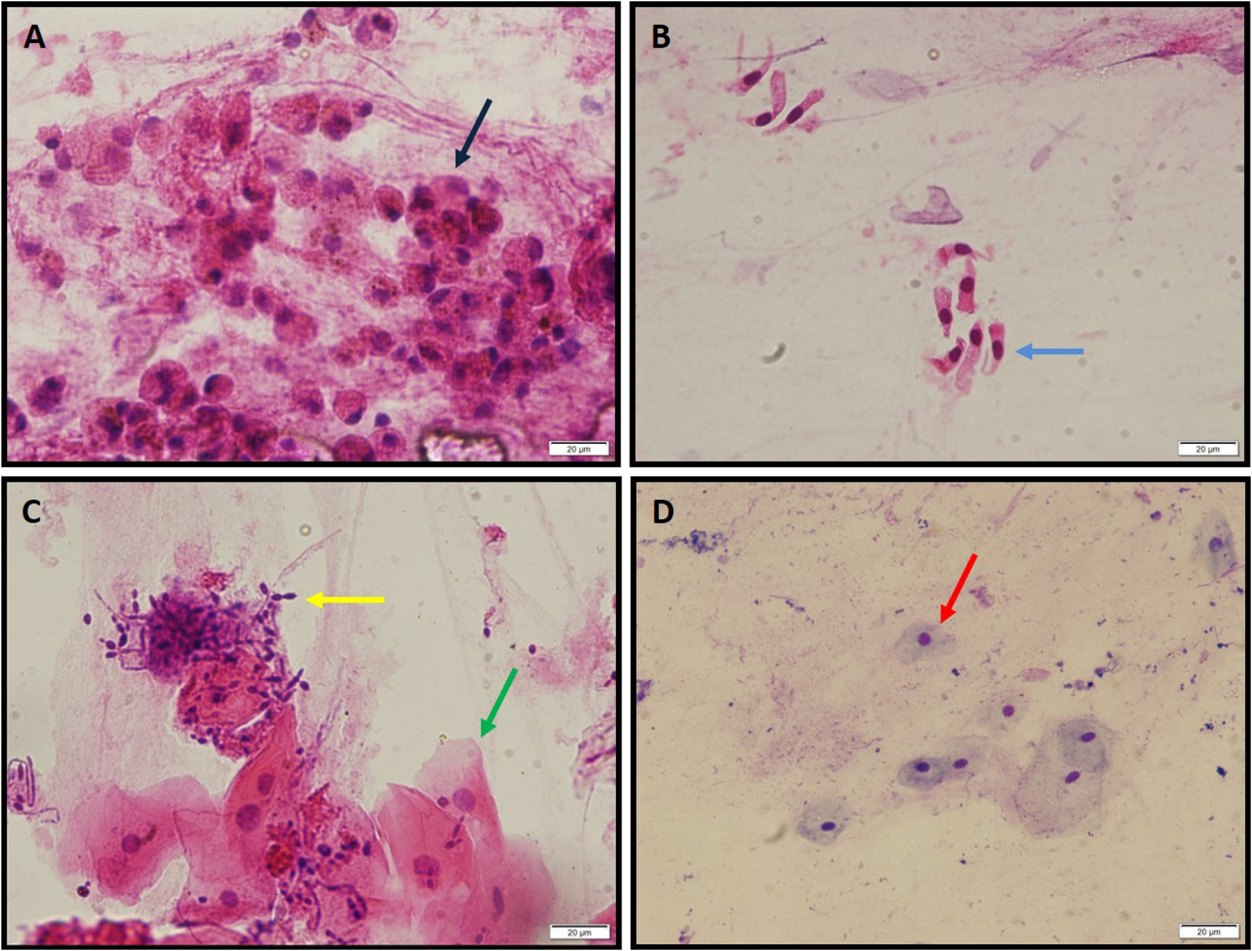
Histopathological examination of sputum samples (hematoxylin and eosin ×20), (a and b) Sputum sample with sufficient criteria, black arrow: alveolar histiocyte, blue arrow: bronchial epithelial cell. (c and d) Insufficient cytological material, oropharyngeal content, yellow arrow: candida hyphae and spores of oral flora, green and red arrow: squamous epithelial cells of the oral mucosa.

Sputum samples of 47 patients were evaluated. Of these, 24 samples belonged to group R and 23 belonged to group C. Among these, 28 samples were evaluated as sufficient, of which 18 belonged to group R and 10 to group C. In light of these findings, it was observed that the rate of patients who could produce sufficient sputum was 43% in group C, whereas the same rate was 75% in group R (Figure 3).

### Microbiological Evaluation

In group R, the most common agent was *Streptococcus* spp. in 11 culture samples. The number of patients with the presence of differently detected bacterial species is listed as follows: *Klebsiella pnuemoniae* in 6 patients; *Enterococcus feacium* and *Streptococcus pnuemoniae* in 3; *Escherichia coli*, *Staphylococcus aureus*, and Enterobacter spp. in 2; *Acinetobacter boumannii*, *Hafnia alvei*, *Rothia* spp., and *Burkholderia* spp. growth were observed in only 1 patient.

In group C, sputum evaluation of 22 patients revealed *Streptococcus pygenes* growth in 2 patients, whereas all other detected microbes were oral flora.

## Discussion

This study demonstrated that pulmonary rehabilitation applied with the HFCWO device in patients with COVID-19 in the early period contributed to the improvement of oxygenation by providing significant improvement as observed in the PFTs of the patients.

Although the specific indications of pulmonary rehabilitation for COVID-19 are not completely clear, considering the possible effects and consequences of the disease on the respiratory system, pulmonary rehabilitation in these patients would be inevitably effective.

In patients with COVID-19, pulmonary rehabilitation is believed to be effective in managing dyspnea, cough, respiratory failure, and gas exchange abnormalities during the acute illness period. In the chronic period, it is believed to be effective against fatigue, chronic respiratory symptoms, nutritional deficiency, difficulties in daily life activities due to decrease in functional status, decrease in work performance, deterioration in quality of life, and psychosocial problems.^13^

As there is not enough information about the long-term outcomes after the active period of COVID-19 infection, the extent of persisting damage or sequelae in patients is unclear. Pulmonary rehabilitation interventions will be definitely required in appropriate patients at the appropriate time. Peter Thomas et al^14^ reported that assisted coughing or stimulation of cough could be an effective method of pulmonary rehabilitation in terms of facilitating the clearance of secretions from the lungs of patients with COVID-19. However, there is no consensus in the literature about the time at which pulmonary rehabilitation should be started. One of the goals of pulmonary rehabilitation in general is to reduce airway resistance and improve ventilation by preventing the accumulation of secretions with positioning, mobilization, effective cough, and other secretion drainage methods to keep the airways open.^13^

In this study, we applied pulmonary rehabilitation by means of HFCWO in the early period in patients with acute COVID-19 symptoms including patients in the second and third groups according to the WHO classification.^2^ Pulmonary rehabilitation was applied with standard treatment using the HFCWO method for 5 days from the first day of hospitalization during acute disease. Following treatment, it became easier for the patients to expel sputum, and thus, the respiratory tract resistance was reduced by preventing the accumulation of secretions. The clinical consequence of this finding was improved oxygenation, significant improvements in pulmonary function, and reduced need for NIMV.

Among statistically insignificant categorical data, the need for HFO was two times higher in Group C compared to Group R, whereas the need for IMV was observed in 4 patients in Group C and no patient in Group R, indicating that the rehabilitation method used was reliable and effective. Moreover, it is noteworthy that there was no mortality in the pulmonary rehabilitation group, whereas 3 patients were dead in the control group.

Emphasis has been placed on preventing nosocomial dissemination while performing pulmonary rehabilitation in patients with COVID-19 infection, providing healthcare with personal protective equipment and as quickly as possible, and managing rehabilitation primarily using telemedicine methods.^15,16^

This is the first study in the literature in which pulmonary rehabilitation was performed with a rehabilitation vest using the HFCWO technique. A crucial advantage of this treatment method is that the patient does not need the presence of a healthcare professional during treatment. With this treatment, which is similar to the postdural drainage method performed with traditional manual therapy, bronchial secretion is activated, and its excretion is facilitated. Patient groups in whom bronchial secretion drainage was applied with this technique in the last 10 years include those with neuromuscular diseases, COPD, and cystic fibrosis, and the usefulness of the method has been scientifically proven.^17-20^

Since the last half century, chest wall physiotherapy has been performed by chest wall vibration and manual percussion methods. These methods are also used in the postoperative period to clear pulmonary secretions.^21^ Various studies in the literature have demonstrated that manual physiotherapy is highly beneficial in such patients in the postoperative period;^22,23^ however, manual physiotherapy causes pain and restlessness in patients.^23^ Another disadvantage of manual physiotherapy is that it is dependent on a practitioner, and hence, standardization is difficult.^24^ Furthermore, the manual percussion method is labor-intensive, costly, and causes pain. In comparison, HFCWO method is effective, reliable, and does not cause pain or discomfort to patients.^25^

High-frequency chest wall oscillation therapy mobilizes bronchopulmonary secretions following minimal cough by compressing the thorax wall externally.^8^ In patients with obstructive pulmonary disease, HFCWO treatment improves the reduced functional residual capacity by increasing central and peripheral mucus clearance in the respiratory tract.^26^ In another study conducted on elderly patients who underwent pulmonary rehabilitation for 6 weeks after COVID-19 infection, significant improvements were observed in FEV1 and FVC levels in PFTs.^27^ Consistent with the results of this study, significant improvements were achieved in the present study on the fifth day FEV1, FVC, and PEF values in patients of Group 4 compared to the control group.

In a previous study, HFCWO treatment was applied for 5 days after weaning to patients who underwent prolonged mechanical ventilation, and the daily sputum amount and improvement observed in chest radiography images were examined. A significant increase was observed in these 2 parameters in patients treated with HFCWO compared to the non-HFCWO group.^28^ In the present study, sputum was evaluated qualitatively and not quantitatively after cytopathological examination. Broncho-alveolar cleaning was found to be more effective after pulmonary rehabilitation with HFCWO. According to the results obtained in the present study, cytopathological observation of bronchial epithelial cells, squamous cells, and alveolar histiocytes in sputum samples from Group R showed that sufficient sputum was produced in these patients In the Group C, the examined sputum samples were insufficient, and their contents were mostly oropharyngeal cells. These results show that early respiratory rehabilitation with HFCWO can contribute to the treatment process in patients with COVID-19 by providing effective expectoration.

Microbiological results demonstrated microbial growth in sputum cultures of 26 patients who underwent pulmonary rehabilitation; however, there it was observed only in 2 sputum cultures in 22 patients in the control group. The rest of the cultures in the control group consisted of normal oral flora. This result suggests that superinfection could occur in patients with COVID-19 during the treatment process, and the microbiological examination of the sputum samples obtained during rehabilitation with HFCWO may direct the selection of appropriate complementary antibiotic treatment. This could enable clinicians to perform antibiotic treatment targeted to specific microbial agents.

There are certain limitations in this study. First, pulmonary rehabilitation was applied for 5 days after the patient’s hospitalization, and a longer pulmonary rehabilitation could have affected the results. Second, long-term results of PFT and radiological evaluations of patients who were discharged after recovery could yield important findings. Third, this study was conducted on patients who do not need intensive care during their first hospitalization and who receive inpatient treatment in the ward. PaO_2_/FiO_2_ ratio could not be calculated because arterial blood gas analysis could not be performed in each patient hospitalized in the ward. Oxygenation was evaluated by SpO_2_. Evaluating the PaO_2_/FiO_2_ ratio would have given more reasonable results. Finally, this is the first study conducted with this device in patients with COVID-19, and the number of patients was determined according to the primary purpose of the study, which was the evaluation of pulmonary functions. The efficacy of HFCWO applications based on different parameters can be further evaluated in large patient series.

In conclusion, this study showed that pulmonary rehabilitation using the HFCWO device applied in patients with COVID-19 in the early period contributed to the improvement of oxygenation by providing significant improvement in the respiratory function tests of the patients and reduced the need for NIMV in the patients. We believe that the HFCWO method will vastly contribute to the treatment process of the patients, as it is easy to apply, does not require additional health personnel supervision or passive patient effort, and does not cause discomfort and pain.

## Figures and Tables

**Table 1.** Demographic Characteristics of Study Patients

	Control Group (n = 50)	Rehabilitation Group (n = 50)	*P*
**Age (years)**	55.86 ± 13.70	57.08 ± 13.62	.754^a^
**Weight (kg)**	82.09 ± 13.87	81.84 ± 11.59	.825^a^
**Height (cm)**	167.86 ± 8.80	168.70 ± 9.25	.643^b^
**Sex (M/F)**	30/20	35/15	.295^c^
**Chronic disease (yes/no)**	22/28	28/22	.230^c^
**Respiratory rate (min–1)**	18.16 ± 3.96	19.40 ± 4.40	.164^a^
**Heart rate**	93.32 ± 13.40	89.52 ± 15.67	.219^a^
**Systolic blood pressure**	135.82 ± 15.92	133.98 ± 16.48	.465^a^
**Diasystolic blood pressure**	83.48 ± 14.37	79.52 ± 10.32	.193^a^

Values are presented as number or mean ± standard deviation.

^a^Mann–Whitney *U*; ^b^Independent samples *t* test; ^c^Pearson chi-square.

**Table 2.** Complete Blood Count and Biochemical Parameters in the Study Patients

		Control Group (n = 50)	Rehabilitation Group (n = 50)	*P*
**First day**	**White blood cells(1/µL)**	7165.44 ± 4256.51	7899.02 ± 4323.07	.228^b^
	**Lymphocyte (1/µL)**	1314.40 ± 650.28	1193.80 ± 719.69	.160^b^
	**Neutrophil (1/µL)**	4945.40 ± 4446.18	5703.16 ± 4188.40	.140^b^
	**HGB (g/dL)**	13.99 ± 2.57	14.22 ± 1.65	.841^b^
	**Platelet (10** **3** **/µL)**	212.72 ± 61.75	238.10 ± 82.45	.103^b^
	**Na (mmol/L)**	138.90 ± 3.19	137.80 ± 3.06	.081^a^
	**K (mmol/L)**	4.24 ± 0.54	4.15 ± 0.44	.328^a^
	**BUN (mg/dL)**	20.54 ± 5.18	22.38 ± 16.59	.624^b^
	**Cr (mg/dL)**	0.90 ± 0.20	0.89 ± 0.26	.897^a^
	**AST (U/L)**	40.62 ± 20.33	38.36 ± 25.34	.248^b^
	**ALT (U/L)**	36.68 ± 21.79	47.98 ± 49.07	.508^b^
	**LDH (U/L)**	328.18 ± 140.05	353.22 ± 166.59	.542^b^
	**Fibrinogen (mg/dL)**	491.08 ± 180.84	520.76 ± 170.97	.401^a^
	**Troponin (ng/L)**	4.50 ± 5.75	4.56 ± 5.18	.471^b^
	**CRP (mg/L)**	42.12 ± 38.45	58.09 ± 46.41	.079^b^
	**Ferritin (ng/mL)**	375.83 ± 441.47	533.69 ± 534.69	.116^b^
**d** **-Dimer (ng/mL)**	788.18 ± 629.54	958.00 ± 925.53	.239^b^
**Fifth day**	**White blood cells (1/µL)**	8398.16 ± 3624.64	9245.50 ± 5393.69	.571^b^
	**Lymphocyte (1/µL)**	1194.49 ± 765.70	1211.40 ± 564.85	.356^b^
	**Neutrophil (1/µL)**	6679.90 ± 3500.59	7554.82 ± 5243.27	.576^b^
	**HGB (g/dL)**	13.80 ± 1.46	14.30 ± 1.48	.094^a^
	**Platelet (10** **3** **/µL)**	272.44 ± 108.67	310.96 ± 116.16	.111^b^
	**Na (mmol/L)**	138.08 ± 6.05	136.40 ± 3.56	.100^b^
	**K (mmol/L)**	4.24 ± 0.48	4.25 ± 0.54	.875^b^
	**BUN (mg/dL)**	21.41 ± 7.59	23.46 ± 9.05	.344^b^
	**Cr (mg/dL)**	0.81 ± 0.21	0.80 ± 0.27	.379^b^
	**AST (U/L)**	38.43 ± 19.04	41.52 ± 31.81	.919^b^
	**ALT (U/L)**	53.04 ± 39.60	61.80 ± 44.75	.247^b^
	**LDH (U/L)**	293.00 ± 119.11	320.18 ± 127.61	.179^b^
	**Fibrinogen (mg/dL)**	458.02 ± 126.77	415.16 ± 107.06	.076^a^
	**Troponin (ng/L)**	8.84 ± 27.24	12.49 ± 59.91	.634^b^
	**CRP (mg/L)**	38.07 ± 59.04	27.04 ± 58.27	.311^b^
	**Ferritin (ng/mL)**	535.20 ± 572.78	528.19 ± 486.25	.759^b^
**d** **-Dimer (ng/mL)**	636.95 ± 598.6	894.30 ± 791.6	.111^b^

Values are presented as mean ± standard deviation.

Na, sodium; K, potassium; BUN, blood urine nitrogen; Cr, creatinine; AST, aspartate aminotransferase; ALT, alanine transaminase; LDH, lactate dehydrogenase; CRP, C-reactive protein.

^a^Independent samples *t* test;^ b^Mann–Whitney *U* test.

**Table 3.** Oxygenation of Study Patients

		Control Group (n = 50)	Rehabilitation Group (n = 50)	*P*
**First day**	**SpO** **2** ** (room air)**	84.54 ± 4.79	80.48 ± 10.41	.232^a^
**SpO** **2** ** (2-4 L/min oxygen)**	90.88 ± 5.06	88.94 ± 6.80	.067^a^
**Fifth day**	**SpO** **2** ** (room air)**	86.45 ± 5.93^*^	86.36 ± 7.77β	.248^a^
**SpO** **2** ** (2-4 L/min oxygen)**	92.51 ± 4.38α	93.26 ± 3.99γ	.335^a^

Values are presented as mean ± standard deviation.

^a^Mann–Whitney *U* test, Wilcoxon sign ranks test for intragroup evaluations.

^*^Compared with first and fifth day SpO_2_ (room air) in the control group, *P* = .003.

^α^Compared with first and fifth day SpO_2_ (with oxygen) in the control group, *P* = .001.

^β^Compared with first and fifth day SpO_2_ (room air) in the rehabilitation group, *P* < .001.

^γ^Compared with first and fifth day SpO_2_ (with oxygen) in the rehabilitation group, *P* < .001.

SpO_2_, oxygen saturation.

**Table 4.** Fifth Day to First Day SpO_2_ Difference Between Groups

	Control Group (n = 50)	Rehabilitation Group (n = 50)	*P*
**Fifth day to first day SpO** **2** ** difference** (room air)	1.82 ± 4.01	5.88 ± 7.20	**.002** **a**
**Fifth day to first day SpO** **2** ** difference** (2-4 L/min oxygen)	1.49 ± 2.90	4.32 ± 5.31	**.001** **a**

Values are presented as mean ± standard deviation.

^a^Mann–Whitney *U* test.

**Table 5.** Pulmonary Function Tests of Study Patients

		Control Group (n = 50)	Rehabilitation Group (n = 50)	*P*
**First day**	**FEV1 (L)**	2.32 ± 0.78	2.54 ± 1.00	0.223^a^
	**FVC (L)**	2.61 ± 0.83	2.90 ± 1.18	0.148^a^
	**FEV1/FVC (%)**	88.32 ± 8.54	86.96 ± 9.58	0.524^b^
**PEF (L/min)**	4.62 ± 1.70	5.47 ± 2.61	0.056^a^
**Fifth day**	**FEV1 (L)**	2.36 ± 0.75	2.72 ± 0.99^*^	**0.045** **a**
	**FVC (L)**	2.56 ± 0.81	3.18 ± 1.16α	**0.003** **a**
	**FEV1/FVC (%)**	90.12 ± 9.22	85.15 ± 9.63	**0.003** **b**
**PEF (L/min)**	4.83 ± 1.89	5.99 ± 2.79β	**0.018** **a**

Values are presented as mean ± standard deviation.

^a^Independent samples *t* test, ^b^Mann–Whitney *U*, Wilcoxon sign ranks test for intragroup evaluations.

^*^Compared with first and fifth day FEV1 in the rehabilitation group, *P* = .011;^ α^Compared with first and fifth day FVC in the rehabilitation group, *P* = .002;^ β^Compared with first and fifth day PEF in the rehabilitation group, *P* = .017.

FEV1, forced expiratory volume in 1 second; FVC, forced vital capacity; PEF, peak expiratory flow.

**Table 6.** Characteristic of Mechanical Ventilation and Status of Patients

	Control Group (n = 50)	Rehabilitation Group (n = 50)	*P*
**HFO (Y/N)**	10/40	5/45	.161^a^
**NIMV (Y/N)**	10/40	2/48	.028^b^
**IMV (Y/N)**	4/46	0/50	.117^b^
**Exitus (Y/N)**	3/47	0/50	.242^b^
**Hospital length of stay (day)**	14.00 ± 5.99	12.70 ± 4.98	.243^c^

Values are presented as number or mean ± standard deviation.

HFO, high-flow oxygen; NIMV, non-invesive mechanical ventilation; IMV, invesive mechanical ventilation; Y, yes; N, no.

^a^Pearson chi-square;^ b^Fisher’s exact test;^ c^Independent samples *t* test.
